# Genome-Wide Analysis of Off-Target CRISPR/Cas9 Activity in Single-Cell-Derived Human Hematopoietic Stem and Progenitor Cell Clones

**DOI:** 10.3390/genes11121501

**Published:** 2020-12-13

**Authors:** Richard H. Smith, Yun-Ching Chen, Fayaz Seifuddin, Daniel Hupalo, Camille Alba, Robert Reger, Xin Tian, Daisuke Araki, Clifton L. Dalgard, Richard W. Childs, Mehdi Pirooznia, Andre Larochelle

**Affiliations:** 1 Cellular and Molecular Therapeutics Branch, National Heart, Lung and Blood Institute, National Institutes of Health, Bethesda, MD 20892, USA; smithr@nhlbi.nih.gov (R.H.S.); robert.reger@nih.gov (R.R.); daisuke.araki@nih.gov (D.A.); childsr@nhlbi.nih.gov (R.W.C.); 2 Laboratory of Bioinformatics and Computational Biology, National Heart, Lung, and Blood Institute, National Institutes of Health, Bethesda, MD 20892, USA; yun-ching.chen@nih.gov (Y.-C.C.); fayaz.seifuddin@nih.gov (F.S.); mehdi.pirooznia@nih.gov (M.P.); 3 The American Genome Center, Uniformed Services University of the Health Sciences, Bethesda, MD 20814, USA; daniel.hupalo.ctr@usuhs.edu (D.H.); camille.alba.ctr@usuhs.edu (C.A.); clifton.dalgard@usuhs.edu (C.L.D.); 4 Office of Biostatistics Research, National Heart, Lung, and Blood Institute, National Institutes of Health, Bethesda, MD 20814, USA; tianx@nhlbi.nih.gov

**Keywords:** CRISPR/Cas9 (clustered regularly interspaced short palindromic repeats/CRISPR-associated protein 9), off-target activity, whole genome sequencing (WGS)

## Abstract

CRISPR/Cas9 (clustered regularly interspaced short palindromic repeats/CRISPR-associated protein 9)-mediated genome editing holds remarkable promise for the treatment of human genetic diseases. However, the possibility of off-target Cas9 activity remains a concern. To address this issue using clinically relevant target cells, we electroporated Cas9 ribonucleoprotein (RNP) complexes (independently targeted to two different genomic loci, the *CXCR4* locus on chromosome 2 and the *AAVS1* locus on chromosome 19) into human mobilized peripheral blood-derived hematopoietic stem and progenitor cells (HSPCs) and assessed the acquisition of somatic mutations in an unbiased, genome-wide manner via whole genome sequencing (WGS) of single-cell-derived HSPC clones. Bioinformatic analysis identified >20,000 total somatic variants (indels, single nucleotide variants, and structural variants) distributed among Cas9-treated and non-Cas9-treated control HSPC clones. Statistical analysis revealed no significant difference in the number of novel non-targeted indels among the samples. Moreover, data analysis showed no evidence of Cas9-mediated indel formation at 623 predicted off-target sites. The median number of novel single nucleotide variants was slightly elevated in Cas9 RNP-recipient sample groups compared to baseline, but did not reach statistical significance. Structural variants were rare and demonstrated no clear causal connection to Cas9-mediated gene editing procedures. We find that the collective somatic mutational burden observed within Cas9 RNP-edited human HSPC clones is indistinguishable from naturally occurring levels of background genetic heterogeneity.

## 1. Introduction

Hematopoietic stem and progenitor cells (HSPCs) are important targets of gene-based regenerative therapies [1]. Permanent correction of inherited hematologic, metabolic and immunologic disorders can be achieved owing to the remarkable ability of long-term repopulating HSPCs to reconstitute and maintain a functional hematopoietic system in vivo. Unequivocal clinical benefits have been obtained over the past decade by vector-mediated addition of a therapeutic gene to HSPCs using replication-defective integrating retroviruses [2]. In recent years, transformative technological advances have allowed the precise editing of cellular genomes, potentially obviating concerns regarding insertional mutagenesis inevitably associated with integrating vectors, as well as enabling appropriate transgene expression levels from endogenous cellular promoters and extending gene therapies to disorders requiring genetic correction of abnormal gene products rather than simple gene addition [2,3,4].

The type II clustered, regularly interspaced, short palindromic repeats (CRISPR) and CRISPR-associated protein 9 (Cas9) bacterial adaptive immune system, known as CRISPR/Cas9, has gained widespread acceptance as a programmable genome editing tool by offering simplicity and flexibility for gene editing applications. The CRISPR/Cas9 system of *Streptococcus pyogenes* (SpCas9) is currently the most popular CRISPR/Cas-derived system, but several orthologs of Cas9 have been developed recently that offer application-specific advantages (such as reduced nuclease size) and expand the accessible repertoire of potential target sites [5,6,7,8]. For genome editing experiments, the Cas9 endonuclease can be easily directed to a cognate target sequence by provision of a small RNA molecule of approximately 100 nucleotides in length, known as the single guide RNA (sgRNA) [9]. The Cas9 protein forms a ribonucleoprotein (RNP) complex with the sgRNA and, upon recognition of an appropriate DNA target sequence, creates a double-stranded break (DSB) within the DNA duplex. For DNA cleavage activity to occur, the target site must be located immediately upstream of a short ancillary sequence known as the protospacer adjacent motif (or PAM sequence) which, in the case of SpCas9, is a 5′-NGG-3′ triplet (where N is any nucleotide) [9]. In the absence of a homologous DNA donor template, the DNA DSB is rapidly repaired by the cellular non-homologous end joining (NHEJ) pathway, often resulting in small insertions and deletions (indels) that can disrupt protein encoding open reading frames. Alternatively, if an appropriate donor template is available, the DNA DSB may be repaired by the homology-directed repair (HDR) pathway [4].

In therapeutic contexts, unintended Cas9-mediated DNA breaks at genomic positions with imperfect complementarity to the guide sequence (off-target sites) could permanently alter normal gene expression and function, and facilitate unpredictable clinical complications. Thus, nuclease precision is imperative. As a consequence, substantial effort has gone into quantifying Cas9-mediated off-target activity in various cellular systems and model organisms. Putative off-target sites may be computationally predicted by virtue of the number, type and position of mismatches relative to the target sequence [10,11]. Additionally, a palette of increasingly sensitive and unbiased off-target detection methods has recently emerged, based on capture of DNA DSB formation in cells (LAM-HTGTS [12], GUIDE seq [13], IDLV capture [14], VIVO [15], DISCOVER-seq [16]), in situ (BLISS [17], BLESS [18]), in vitro (CIRCLE-seq [19], Digenome-seq [20], SITE-seq [21]) or by CHIP-seq as a proxy for genome-wide cleavage events [22]. However, each approach has its own intrinsic biases. In contrast, whole genome sequencing (WGS) provides a broad-based method to assess the extent of Cas9-mediated off-target activity in gene-edited cells and tissues, including indels, single nucleotide variants (SNVs) and structural variants (SVs) such as inversions, rearrangements, duplications and large deletions. With decreasing sequencing costs, this approach has gained traction in recent years and shown utility for assessment of off-target mutations caused by Cas9 in human cells [20,23,24,25,26,27], mice [28,29,30,31,32,33,34,35,36], rats [28], goats [37] and plants [38].

Ample evidence of Cas9 promiscuity in human transformed cell lines initially raised concerns over the therapeutic promise of engineered nuclease platforms [14,23,39,40,41,42,43,44,45,46]. These studies provided the impetus to develop new strategies that mitigate the risk of Cas9 off-target cleavage. Delivering Cas9/sgRNA as an RNP complex was shown to increase editing specificity by limiting the duration of exposure to the CRISPR reagents [47,48]. Chemical modification of synthetic sgRNA can similarly reduce off-target effects, perhaps by destabilizing imperfect sgRNA/target heteroduplexes [47,49]. Cas9 mutants with improved specificity have also been generated using approaches based on bacterial genetic screens or rational mutagenesis of contact residues between Cas9 and the targeted sequence [50,51,52,53,54,55,56].

Genome editing with CRISPR/Cas9 has entered clinical trials for the treatment of a variety of diseases, many of them hematological in nature, and a number of additional studies targeting human HSPCs are poised to follow [4]. However, Cas9 precision in these cells has not been fully investigated using unbiased approaches and clinically relevant genome editing procedures. Accordingly, we performed WGS analysis to obtain a genome-wide, unbiased characterization of potential off-target Cas9 activity in peripheral blood-mobilized human HSPC clones that had undergone genome editing at one of two independent genetic loci via electroporation of Cas9/sgRNA RNP complexes.

## 2. Materials and Methods

### 2.1. Human HSPC Isolation

Human CD133+ HSPCs were obtained from a female healthy donor after informed consent in accordance with the Declaration of Helsinki, under an Institutional Review Board-approved clinical protocol (NCT00082329) and shared with our laboratory without identifiers. Briefly, HSPCs were mobilized to the peripheral circulation by subcutaneous injection of granulocyte-colony stimulating factor for 5 consecutive days, with an additional injection of AMD3100 (plerixafor) given on day 4. HSPCs were isolated by leukapheresis and subsequent enrichment by CD133-specific immunoaffinity selection. Cells were stored under liquid nitrogen vapor phase until use. De-identified HSPC samples were processed for WGS analysis as described below.

### 2.2. Electroporation of Human HSPCs

Prior to electroporation, primary human HSPCs were cultured for 48 h in StemSpan SFEM II serum-free culture medium (StemCell Technologies, Inc., Cambridge, MA, USA) supplemented with 100 U/mL penicillin, 100 µg/mL streptomycin, and 100 ng/mL each of the following cytokines (from PeproTech, Inc., Cranbury, NJ, USA): thrombopoietin (TPO), stem cell factor (SCF) and FMS-like tyrosine kinase 3 ligand (Flt3-L). For electroporation, individual HSPC samples (~3.5 × 10^6^ cells) were aliquoted into sterile microfuge tubes and pelleted at 3000 rpm for 5 min in a Sorvall Legend Micro 17 centrifuge (Thermo Fisher Scientific, Waltham, MA, USA). Cell pellets were resuspended in 100 µL MaxCyte electroporation buffer (MaxCyte, Inc., Gaithersburg, MD, USA) and electroporated (with or without Cas9 or Cas9 RNPs) in OC-100 Processing Assemblies (MaxCyte) using a MaxCyte GT electroporator and program HPSC34-3-OC. For gene editing, 20 µg of purified recombinant SpCas9 protein (PNA Bio, Inc., Thousand Oaks, CA, USA) was pre-complexed on ice for 20 min with 10 µg of the appropriate chemically-modified sgRNA (bearing 2′-O-methyl phosphorothioate-modified nucleotides at the first 3 and last 3 positions of the synthetic sgRNA) prior to addition to the appropriate sample. The cognate 20-mers incorporated into the individual sgRNAs were as follows: AAVS1, 5′-GUUAAUGUGGCUCUGGUUCU-3′ and CXCR4, 5′-UCUUCUGGUAACCCAUGACC-3′. “Cas9 only” samples received 20 ug SpCas9 in the absence of sgRNA. Electroporated cells were allowed to rest for 20 min at 37 °C and then transferred to 10 cm tissue culture dishes containing 10 mL of StemSpan SFEM II medium with supplements and incubated overnight at 37 °C in a 5% CO_2_ atmosphere prior to single-cell cloning.

### 2.3. Single-Cell Cloning

Gene-edited human HSPCs and controls were cloned in 96-well tissue culture plates by limiting dilution. Briefly, electroporated cells were stained with acridine orange-propidium iodide (AOPI) to determine viability and enumerated using a Nexcelom Cellometer (Nexcelom Bioscience, Lawrence, MA, USA). Viable cells were then diluted in StemSpan SFEM II tissue culture medium (supplemented with 100 U/mL penicillin; 100 µg/mL streptomycin; and 100 ng/mL of TPO, SCF and Flt3-L) to a concentration of 1 cell per 200 µL medium. Diluted cells were distributed into 96-well tissue culture plates (200 µL medium per well). Individual wells were inspected by bright-field microscopy to confirm the presence of a single cell. Ninety-six well plates were incubated in a humidified 37 °C, 5% CO_2_ incubator under normoxic conditions for up to 4 weeks prior to genomic DNA isolation.

### 2.4. DNA Isolation

Genomic DNA was isolated from bulk HSPC samples (~2 × 10^6^ cells per sample) using a QIAGEN Blood & Tissue DNA isolation kit (QIAGEN, Inc., Germantown, MD, USA) as per the manufacturer’s instructions. For HSPC clonal isolates, genomic DNA was extracted using a NucleoSpin Tissue XS kit (TAKARA Bio USA, Inc., Mountain View, CA, USA) following the manufacturer’s protocol with the modification that cellular colonies were harvested in-well in 80 µL of kit-supplied buffer T1 per clonal isolate before transfer to individual microfuge tubes for further processing. Purified genomic DNA was eluted into low DNA-binding microcentrifuge tubes (SafeSeal, Sorenson BioScience, Inc., Salt Lake City, UT, USA).

### 2.5. T7 Endonuclease I Assay

Cas9 target sites within the *CXCR4* and *AAVS1* loci were PCR amplified in a 50 µL reaction volume per sample using high-performance RANGER PCR Mix (Bioline USA, Inc., Memphis, TN, USA), 1 ng of genomic DNA and 100 ng each of the appropriate forward and reverse primer. Target-specific primer sets were: CXCR4-F 5′-AGAAAGCAAGCCTGAATTGG-3′; CXCR4-R, 5′-GGATGGGGTTCAGACAACAG-3′; AAVS1-F, 5′-CTTGCTTTCTTTGCCTGGAC-3′; AAVS1-R, 5′-ACACCTAGGACGCACCATTC-3′. PCR parameters were as follows: 95 °C for 3 min, followed by 35 rounds of 98 °C for 15 s, 55 °C for 30 s, 72 °C for 2 min, with a final extension step at 72 °C for 5 min. After amplification, each PCR reaction was partially purified using a BioRad PCR Kleen column (BioRad Laboratories, Hercules, CA, USA) according to the manufacturer’s instructions. T7 endonuclease I (T7E1) reactions were assembled in 8-well, 0.2 mL PCR strips with ≤200 ng of column-purified PCR product per reaction. Amplicons were heat-denatured and re-annealed in a programmable thermocycler using the following hybridization conditions: 95 °C for 5 min, ramp from 95 to 85 °C at −2 °C per second, ramp from 85 to 25 °C at −0.1 °C per second, followed by a “hold” at 22°C. After re-annealing, 10 units of T7E1 (New England Biolabs, Inc., Ipswich, MA) were added to the appropriate tubes, and the samples were incubated at 37 °C for 15 min. After T7E1 digestion, 4 µL of NOVEX™ 5X Tris-borate-EDTA (TBE) gel loading buffer was directly added to each PCR tube. A portion of each sample was loaded onto a pre-cast Novex™ 4–20% polyacrylamide TBE gel (Thermo Fisher Scientific), followed by electrophoresis at 150 volts (constant voltage) until the lower tracking dye approached the bottom of the gel.

### 2.6. NGS Library Preparation and High-Throughput Illumina Sequencing

Samples were entered into the seqEngine Laboratory Information Management System for generation of a unique project and sample barcode tracking number linked to sample- and subject-level data. Visual QC of received tubes and samples was conducted, followed by DNA quantitation utilizing a fluorescent dye-based assay (PicoGreen dsDNA reagent; Thermo Fisher Scientific, Waltham, MA, USA) with measurement on a SpectraMax Gemini XS microplate reader (Molecular Devices, San Jose, CA, USA). Sequencing libraries were generated from 1–10 ng of genomic DNA using the Illumina Nextera DNA Flex Library Prep Kit (Illumina, Inc., San Diego, CA, USA) using the standard protocol with 12 cycles of amplification. Nextera DNA CD Index adapters (96 indexed, 96 samples) for ligation were used in workflow. Library size distribution and absence of free adapters or adapter dimers was assessed by automated capillary gel-electrophoresis (Advanced Analytical Fragment Analyzer; Agilent Technologies, Inc., Santa Clara, CA, USA ). Library yield was determined by qPCR quantitation using the KAPA qPCR Quantification Kit (Roche Light Cycler 480 Instrument II). After qPCR quantitation, libraries were normalized to 2.2 nM in a working 96-well plate by automation (Hamilton STAR Liquid Handling System; Hamilton Company, Reno, NV, USA). Libraries were clustered as 8-plex pools or individually on an Illumina cBot2 using the HiSeq X PE Cluster Kit and a HiSeq X Flow Cell v2.5 before sequencing on an Illumina HiSeq X System with 151+7+151 cycle parameters using HiSeq X HD SBS Kit reagents. Sequencing output BCL files were processed using the Illumina HiSeq Analysis Software v2.0. FASTQ files were generated using the generateFASTQ HiSeq Analysis Workflow.

### 2.7. Bioinformatic Analysis

FASTQ files were aligned to human genome reference build GRCh38 using BWA-MEM aligner (v0.7.17) [57] followed by Picard (v2.20.8) MarkDuplicates (Picard toolkit: https://broadinstitute.github.io/picard/) to identify and mark PCR duplicate reads. GATK (v3.8-1) [58] was employed to perform base quality score recalibration (BQSR) in order to minimize systematic errors and improve downstream genotyping accuracy [59]. Single nucleotide variants and small indels were identified using LoFreq (v2.1.3.1) [60] somatic variant caller algorithms. Somatic variant calls produced by the LoFreq algorithms were integrated to generate aggregate calls for each group using BCFtools [61]. The final LoFreq call set was filtered for autosomal chromosomes (Chr1-22) and ChrX. Genomic structural variants were identified using three structural variant callers, BreakDancer (v1.4.5) [62], Manta (v1.6.0) [63], and Delly (v0.8.3) [64] and merged using Survivor (StructURal Variant majorIty VOte) (v.1.0.7) Software Tool [65] to determine structural variants that were shared among these three callers.

### 2.8. Data Availability

Sequencing data were deposited in NCBI Sequence Read Archive (SRA, https://www.ncbi.nlm.nih.gov/sra/) under BioProject accession number PRJNA680910.

### 2.9. Statistical Analysis

The Kruskal–Wallis test was used to compare the somatic indel frequency and somatic SNV frequency observed among the Cas9 RNP-recipient and control treatment groups. This nonparametric rank-based method tests the null hypothesis that the median number of variants is the same among the five treatment groups (i.e., “No EP”, “EP only”, “Cas9 only”, “CXCR4 RNP” and “AAVS1 RNP”). If the results of the Kruskal–Wallis test were significant, then Dunn’s multiple comparison test was used to compare the mean differences between the treatment groups and the No EP control group. All tests were two-sided. A *p*-value less than 0.05 was considered statistically significant.

## 3. Results

### 3.1. Genome-Wide Analysis of Cas9 Promiscuity in Single-Cell-Derived Human HSPC Clones

**Figure 1:**
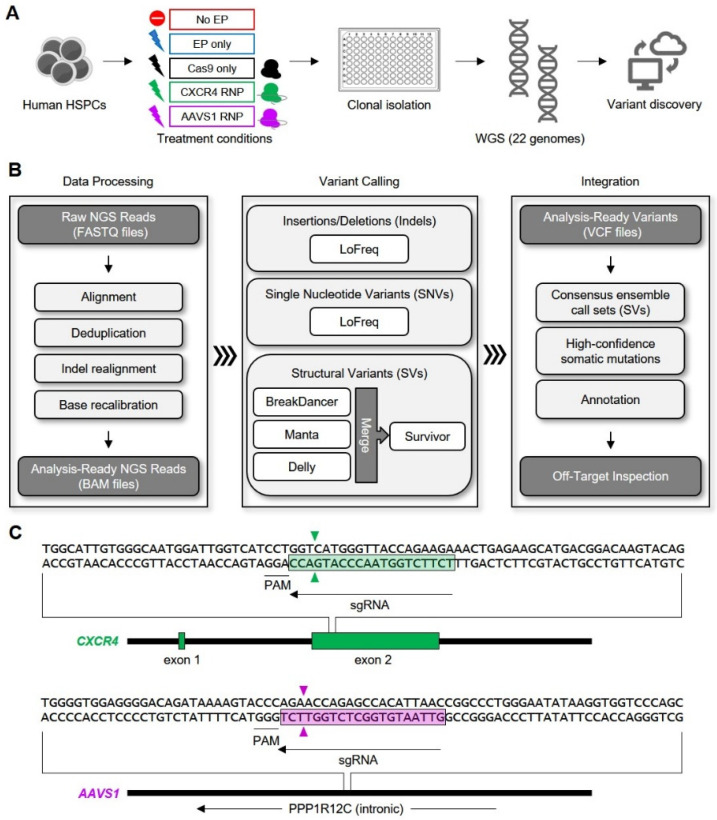
Genome-wide analysis of off-target CRISPR/Cas9 (clustered regularly interspaced short palindromic repeats/CRISPR-associated protein 9) activity in human hematopoietic stem and progenitor cells (HSPCs). (**A**) Experimental design. Human HSPCs were either not electroporated (“No EP”), electroporated in the absence of Cas9 ribonucleoprotein (RNP) complexes (“EP only”), electroporated with purified Cas9 protein in the absence of single guide RNA (sgRNA) (“Cas9 only”) or electroporated with the indicated Cas9 RNP complex (“CXCR4 RNP” or “AAVS1 RNP”). Clonal isolates were subjected to high-throughput whole genome sequencing analysis. (**B**) Bioinformatics pipeline. Phase 1—Data processing: Reads were aligned to human genome reference build GRCh38 using the Burrows-Wheeler Aligner (BWA-MEM) algorithm. Analysis-ready BAM files were prepared by utilizing Picard (MarkDuplicates) software to identify and remove PCR duplicates, followed by localized indel realignment and base quality score recalibration. Phase 2—Variant calling: Identification of novel indels (small insertion/deletion mutations) and single nucleotide variants (SNVs) was performed using LoFreq. Structural variants (SVs) were identified using three structural variant callers, Delly, Manta, and BreakDancer. The three call sets were merged using Survivor. Phase 3—Integration: Somatic variant calls were integrated to generate consensus calls for each group using BCFtools. NGS, next generation sequencing. (**C**) Schematic representation of genomic sgRNA target sites. Cas9 RNP cleavage sites are indicated by triangles.

To address the significance of off-target CRISPR/Cas9 activity in human blood stem cells, we performed Cas9 RNP-based genome editing in peripheral blood-mobilized human HSPCs obtained from a healthy donor and characterized the genome-wide accumulation of post-editing somatic mutations using high-throughput WGS analysis of single-cell-derived HSPC clones (Figure 1). To distinguish between naturally occurring spontaneous somatic mutations, potential cell manipulation-induced (i.e., non-Cas9-associated) mutagenesis, and bona fide Cas9-mediated genetic alterations, the experimental design included a parallel set of control manipulations applied to samples of the same bulk HSPC population (Figure 1A). The bioinformatics pipeline used to identify indels, SNVs, and SVs is outlined in Figure 1B. HSPC samples were either (i) not electroporated (“No EP”), (ii) electroporated in the absence of CRISPR/Cas9 effector molecules (“EP only”), (iii) electroporated with recombinant SpCas9 alone (“Cas9 only”) or (iv) electroporated with RNPs consisting of Cas9 bound to 2′-*O*-methyl phosphorothioate-modified sgRNAs. Cas9 RNPs were targeted to either of two genetic loci: exon 2 of the *CXCR4* gene on chromosome 2 (“CXCR4 RNP”), or the insertional “safe harbor” locus, *AAVS1* [66], on chromosome 19 (“AAVS1 RNP”). The sgRNA target sequences are depicted in Figure 1C.

**Figure 2:**
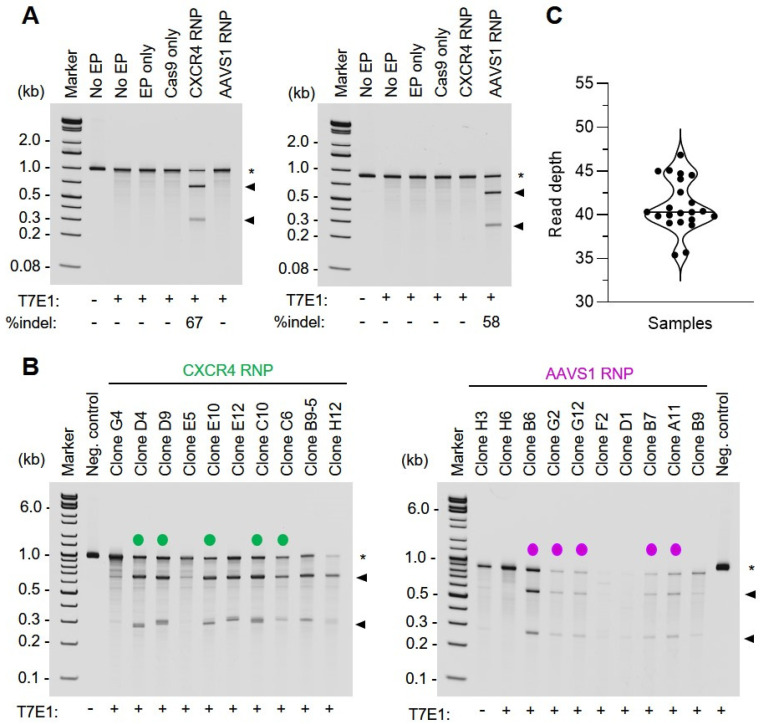
Characterization of CRISPR/Cas9 genome editing by T7 endonuclease I (T7E1) assay. (**A**) T7E1 analysis of CRISPR/Cas9-mediated gene editing in bulk HSPCs. For analysis of bulk (i.e., pre-clonal) HSPC gene editing efficiency, genomic DNA (gDNA) was isolated from gene-edited and control HSPC samples at 3 days post-treatment. Isolated gDNA was subjected to PCR amplification using either *CXCR4* (left panel) or *AAVS1* (right panel) target site-specific primer sets as described in Materials and Methods. Where indicated, PCR amplicons were digested with T7E1, and enzymatic cleavage products were resolved by electrophoresis on a native 4–20% polyacrylamide gradient gel. Amplicon substrates are indicated by an asterisk (*), T7E1 cleavage products are indicated by arrowheads. Clonal isolates selected for WGS and variant analysis are indicated by a color-coded dot. (**B**) T7E1 analysis of CRISPR/Cas9-mediated gene editing in HSPC clonal isolates. For analysis of gene editing efficiency in HSPC clonal isolates, gDNA was extracted from cellular colonies approximately 2–4 weeks post-treatment. Isolated gDNA was subjected to PCR amplification using either *CXCR4* (left panel) or *AAVS1* (right panel) target site-specific primer sets. Where indicated, PCR amplicons were digested with T7E1, and enzymatic cleavage products were resolved by native polyacrylamide gel electrophoresis (4–20% gradient gel). Amplicon substrates are indicated by an asterisk (*), T7E1 cleavage products are indicated by arrowheads. Clonal isolates selected for whole genome sequencing (WGS) and variant analysis are indicated by a color-coded dot. EP, electroporation; kb, kilobase pairs; sgRNA, single guide RNA. (**C**) WGS read depth. Violin plot showing read depth for each sample. The median (40.3×) is indicated by a black line.

RNP-based genome editing in the bulk HSPC population demonstrated a high efficiency of on-target indel formation, with a gene editing efficiency of 67 and 58% at the *CXCR4* and *AAVS1* loci, respectively, as determined by T7 endonuclease 1 (T7E1) assay of target site-derived PCR amplicons (Figure 2A). Following experimental manipulation, cells were cloned by limiting dilution and single-cell isolates were minimally expanded in 96-well plates to obtain sufficient genomic DNA for WGS library preparation and high-throughput sequencing. Limited clonal expansion was utilized to diminish the potential for culture-associated genetic alteration, resulting in the recovery of nanogram amounts of genomic DNA in most instances. Prior to whole genome sequencing, successful gene editing within Cas9 RNP-recipient single-cell clones was confirmed by T7E1 assay of target site-derived PCR amplicons (Figure 2B). High-throughput sequencing of the HSPC samples (3–5 per treatment group plus bulk reference sequence) resulted in approximately two terabytes of data, representing 22 separate human whole genome sequence determinations with a median read depth of ~40× (range from 35× to 47×) (Figure 2C).

### 3.2. Characterization of Indels and SNVs

**Figure 3:**
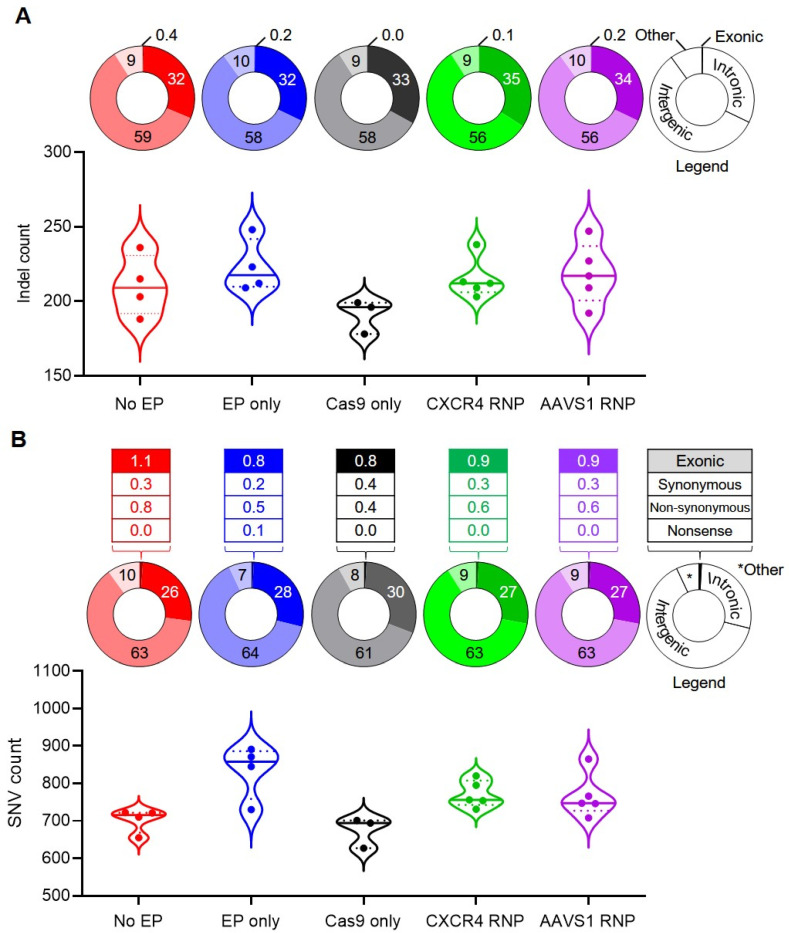
Characterization of non-targeted indels and single nucleotide variants (SNVs). (**A**) The frequency of non-targeted indels is plotted for each treatment group (*n* = 3 to 5). The percentage of non-targeted indels observed within exons, introns, intergenic regions, and other genomic features (e.g., 5′ UTRs, 3′ UTRs, splicing signals, and ncRNAs) for each treatment group is indicated by a donut plot above each group. A nonparametric Kruskal–Wallis test found no statistical difference in the mean indel count among the treatment groups (*p* = 0.1730). (**B**) The frequency of SNVs for each data set is plotted (*n* = 3 to 5). The percentage of SNVs mapping within exons, introns, intergenic regions, and other genomic features (e.g., 5′ UTRs, 3′ UTRs, splicing signals, and ncRNAs) is indicated by a donut plot above each group. Kruskal–Wallis analysis indicated a statistically significant difference among the treatment groups (*p* = 0.0093). Dunn’s multiple comparison test comparing each treatment group to the “No EP” baseline control indicated that the “EP only” group demonstrated a weak statistical difference in number of total SNVs (*p* = 0.0414). A *p*-value less than 0.05 was considered statistically significant.

An allele frequency analysis-based somatic variant calling algorithm (LoFreq) [60] was employed to identify indels (small insertions or deletions of approximately 50 base pairs or less) and SNVs captured within the WGS data set relative to the pre-treatment bulk HSPC reference sequence (Figure 3; Tables S1 and S2). Genome-wide data analysis revealed a large number of non-targeted indels (4474) distributed among all samples from both Cas9 RNP-recipient and non-Cas9 RNP-recipient groups (Figure 3A; Table S1). Targeted indels generated within the gene-edited CXCR4 RNP- and AAVS1 RNP-recipient samples were readily identified (Table S1, “on-target hits” tab). Three of the five CXCR4 RNP-treated clones demonstrated potential multi-allelic on-target indel calls. A nonparametric Kruskal–Wallis statistical test indicated no significant difference (*p* = 0.1730) in the frequency of non-targeted indels among Cas9 RNP-recipient groups and control groups. In all treatment groups, >99% of observed non-targeted indel mutations fell within intergenic, intronic, or other non-protein coding regions of the genome (Figure 3A). Relative percentages of indel incidence within intronic, intergenic, exonic, and “other” annotated sequences (e.g., 5′ UTRs, 3′ UTRs, splicing signals, and ncRNAs) were similar among the treatment groups, with the majority of indels mapping to intergenic regions of the genome (range from 56 to 59% of total).

A combined total of 15,855 SNVs were identified among the five treatment groups (Figure 3B; Table S2). A Kruskal–Wallis statistical test indicated a significant difference in the frequency of somatic SNVs observed among the groups (*p* = 0.0093). Further analysis utilizing Dunn’s multiple comparison test indicated that the “EP only” group demonstrated a statistically significant difference in SNV counts compared to the untreated baseline group (i.e., “No EP”), albeit with weak statistical support (*p* = 0.0414). In all treatment groups, the majority of observed SNVs were intergenic, with <2% of SNVs occurring within annotated coding sequences (Figure 3B); however, when present within an open reading frame, the observed SNVs tended to result in nonsynonymous amino acid changes. Overall, the five treatment groups displayed similar distributions of SNVs among annotation categories, regardless of Cas9 RNP application.

To determine the extent of off-target indel and SNV formation at homologous sgRNA target sites within the genome, we inspected a combined total of 623 in silico-identified [67], potential off-target sites (201 potential *CXCR4* off-target sites and 422 potential *AAVS1* off-target sites) bearing significant homology (up to 4 base mismatches in association with a 5′-NRG-3′ PAM sequence) to the unique 20-mer of the CXCR4 or AAVS1 sgRNAs (see Tables S3 and S4). A 1000 base pair window spanning the first base pair of a given homologous off-target site was queried for called variants. Comparison of total called indel variants to 201 homologous *CXCR4* off-target sites revealed no off-target indels associated with the Cas9 RNP-recipient samples. A single clone (sample S2–27) within the “No EP” group displayed a 1-bp insertion near a potential *CXCR4* off-target site on chromosome 2 (Chr2:212921127, see Table S1, “off-target hits” tab). Similarly, comparison of total called indel variants to 422 homologous *AAVS1* off-target sites revealed no off-target indels associated with Cas9 RNP-recipient samples; however, one clone (sample S2–21) within the “CXCR4 RNP” treatment group displayed a fortuitous 2-base pair deletion within the *AAVS1* target site on chromosome 19 (Chr19:55115807, see Table S1, “off-target hits” tab). Of the >15,000 SNVs distributed among the five treatment groups, only five occurred within, or in proximity to, potential *CXCR4* or *AAVS1* homologous off-target sites (see Table S2, “off-target hits” tab), and only 2 of the 5 SNVs originated from Cas9 RNP-recipient samples.

### 3.3. Characterization of Structural Variants

**Figure 4:**
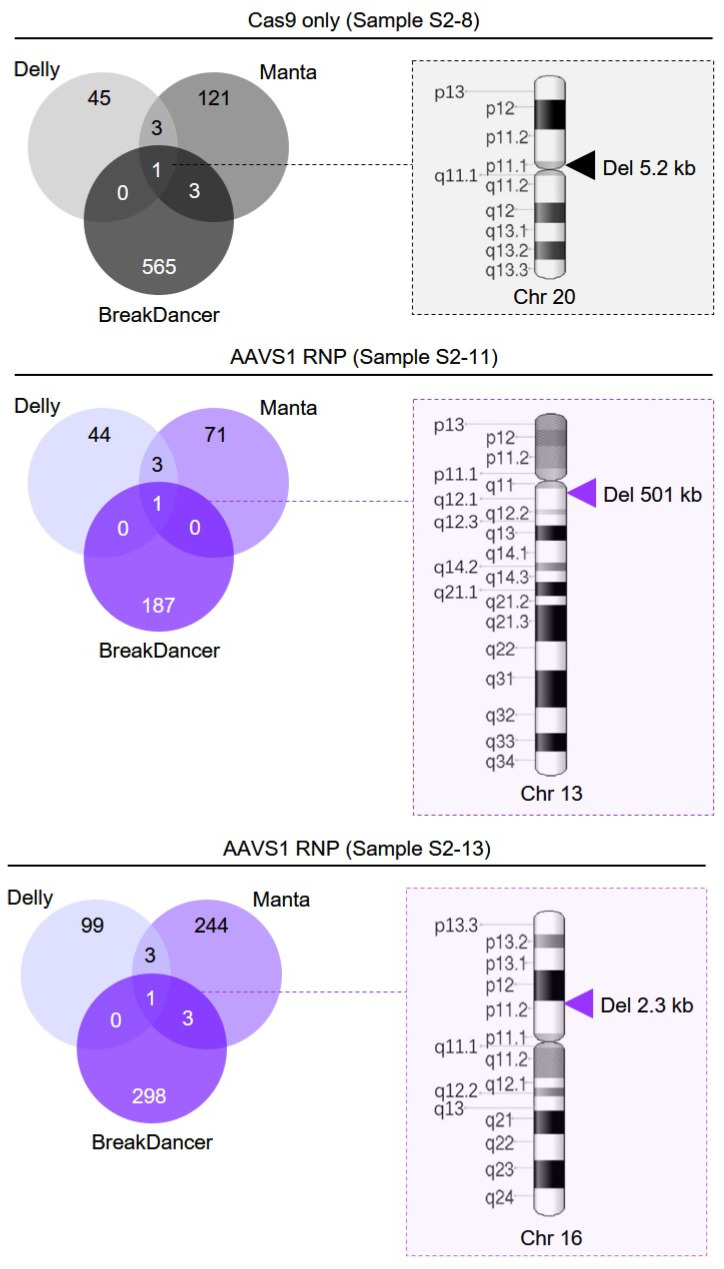
Structural variant (SV) analysis. Venn diagrams representing the intersection of SV calls from three structural variant analysis algorithms, Delly, Manta, and BreakDancer, are depicted for each positive sample from the WGS data set. Schematic diagrams indicating the relative chromosomal location of called SVs for each positive sample are shown. Cytogenetic band designations are indicated at the left of each ideogram. Chromosome image attribution: National Center for Biotechnology Information, U.S. National Library of Medicine/Public domain.

To score high-confidence structural variants, an intersection of call sets generated by three somatic variant calling algorithms (Delly, Manta, and BreakDancer) was used to identify SV mutations among the five treatment groups (Figure 4; Table S5).

Three large deletions—a 501-kb deletion on chromosome 13, a 2.3-kb deletion on chromosome 16 and a 5.2-kb deletion on chromosome 20—were identified. Although two of the deletions were observed within AAVS1 RNP-recipient clones (samples S2–11 and S2–13), neither of the deletions occurred within the AAVS1 RNP-targeted chromosomes (i.e., Chr 19), and none of the called deletion breakpoints mapped in close proximity to potential AAVS1 sgRNA homologous off-target sites. The final structural variant, a deletion of approximately 5.2-kb, was identified on chromosome 20 within one member (sample S2–8) of the “Cas9 only” control group.

## 4. Discussion

In this study, we used WGS to obtain an unbiased, genome-wide evaluation of non-targeted CRISPR/Cas9 activity in primary, human HSPCs independently edited at two different genomic loci. Our analyses indicate that levels of CRISPR/Cas9-mediated off-target mutagenesis associated with electroporation of human blood stem cells with specific Cas9/sgRNA RNPs is essentially indistinguishable from naturally occurring somatic background mutations within recipient cells.

The results of our genome-wide analysis are consistent with most previous studies investigating CRISPR/Cas9 off-target activity in vertebrate embryos and human stem cells. Characterization of unintended mutations in the offspring of mouse [29,32,33,34,35,36,68,69,70], goat [37] or zebrafish [71] zygotes microinjected with Cas9 in combination with sgRNAs targeting several endogenous loci indicated limited or no significant Cas9-attributable increase in observed de novo mutations. A single report suggested a higher degree of Cas9 promiscuity, describing 43 bona fide Cas9-generated off-targets detected by high-coverage WGS of ten Cas9/sgRNA-treated mouse embryos and their genetic parents [28]. In addition to vertebrate embryos, WGS of human pluripotent stem cell clones subjected to CRISPR/Cas9 genome editing also indicated that off-target Cas9-mediated mutations were low frequency events, comparable to levels of sequence variants that accumulate during routine culture [24,25,27]. Our data also cohere with the sequence capture characterization of off-target mutations in a population of nuclease-treated CD34+ HSPCs where indels at a single off-target site were detected for one of five active guides [72]. In contrast, several independent studies examining CRISPR/Cas9 activity in transformed cell lines have reported much higher incidence of cleavage events at unintended sites [14,23,39,40,41,42,43,44,45,46]. The off-target alterations identified in these investigations harbored up to five nucleotide substitutions between the hybridizing sgRNA and the target genomic sequence [42,46], raising concerns with respect to therapeutic applications as frequent sequences with five mismatches to a 20-nucleotide sgRNA may be found in the human genome [39,41].

It is likely that the detected levels of off-target Cas9 activity will differ under distinct experimental conditions. First, the target cells used to evaluate Cas9 precision can influence the observed rate of off-target events. For example, cells with limited division rate, such as human hematopoietic stem cells, may be less susceptible to off-target effect [73]. Unwinding of double stranded DNA during cell division was recently proposed to unmask cryptic off-target sites, thereby facilitating Cas9 recruitment at these previously unexposed genomic loci [73]. This idea is in agreement with genome-wide CHIP-seq [22] and GUIDE-seq [13] analyses reporting increased frequency of off-target mutations in regions of open chromatin. Second, strategies utilized for the cellular delivery of editing components may also have a considerable impact on Cas9 fidelity. In cell lines, most studies have relied on delivery of Cas9 nuclease and sgRNA sequences via plasmid transfection or lentiviral transduction. These approaches produce recombinant Cas9 protein at high levels for prolonged periods of time, thus increasing intracellular concentrations of Cas9 and promoting interaction at low affinity off-target sites that display notably larger dissociation constants (K_d_) relative to the cognate on-target site [41,42]. In primary cells, electroporation of plasmid DNA for coexpression of Cas9 and sgRNA often results in pronounced cytotoxicity, and more lenient approaches based on delivery of nuclease mRNA or Cas9/sgRNA RNP have generally been favored. These methods limit the intracellular half-life, and thus off-target activity, of Cas9/sgRNA complexes while permitting easier control of their cellular concentration for efficient on-target cleavage [47,48]. Third, as the specificity of Cas9-mediated DNA cleavage is locus-dependent and governed by the quantity, position and identity of mismatching bases relative to the targeted site [41,42,46], the choice of sgRNA sequences may significantly influence the occurrence of off-target Cas9 activity within recipient cells. Last, the scope and sensitivity of methods selected for the identification of bona fide off-target sites after genome editing can affect the measured occurrence of unintended mutations. The whole genome sequencing approach used in this report provides a sensitive, genome-wide assessment of nuclease fidelity. A practical drawback is that only a limited number of select clones within a larger population of gene-edited cells can be analyzed by WGS. Additionally, since single-cell HSPC isolates were briefly expanded prior to WGS, off-target genome editing events that abrogate cellular viability or expansion would not be observed; however, such mutations would, by their nature, be self-limiting at the cellular population level and thus present diminished likelihood of contribution to untoward clinical outcomes.

In sum, the aspects described above highlight the need for context-specific evaluation of Cas9 promiscuity. Extrapolation from studies using alternate cell types, editing protocols or off-target detection approaches may not accurately reflect the specificities of RNA-guided nucleases for the intended application. By combining clinically relevant cells and editing procedures with an unbiased genome-wide survey of off-target CRISPR/Cas9 activity in single-cell-derived HSPC clones, our study has relevance to the successful translation of ex vivo, Cas9-based HSPC genome editing strategies to the clinic.

## Supplementary Materials

The following are available online at https://www.mdpi.com/2073-4425/11/12/1501/s1, Table S1: Indel calls; Table S2: SNV calls; Table S3: Homologous *CXCR4* sites; Table S4: Homologous *AAVS1* sites; Table S5: Called SVs.
